# Aggressiveness of Grade 4 Gliomas of Adults

**DOI:** 10.3390/clinpract12050073

**Published:** 2022-09-03

**Authors:** Mariana Deacu, Any Docu Axelerad, Steliana Popescu, Theodor Sebastian Topliceanu, Mariana Aschie, Madalina Bosoteanu, Georgeta Camelia Cozaru, Ana Maria Cretu, Raluca Ioana Voda, Cristian Ionut Orasanu

**Affiliations:** 1 Clinical Service of Pathology, Departments of Pathology, “Sf. Apostol Andrei” Emergency County Hospital, 900591 Constanta, Romania; 2 Faculty of Medicine, “Ovidius” University of Constanta, 900470 Constanta, Romania; 3 Department of Neurology, “Sf. Apostol Andrei” Emergency County Hospital, 900591 Constanta, Romania; 4 Department of Radiology, “Sf. Apostol Andrei” Emergency County Hospital, 900591 Constanta, Romania; 5 Center for Research and Development of the Morphological and Genetic Studies of Malignant Pathology (CEDMOG), “Ovidius” University of Constanta, 900591 Constanta, Romania; 6 Academy of Medical Sciences of Romania, 030167 Bucharest, Romania; 7 Clinical Service of Pathology, Departments of Genetics, “Sf. Apostol Andrei” Emergency County Hospital, 900591 Constanta, Romania

**Keywords:** astrocytoma, CDKN2A, glioblastoma, IDH1, Ki-67

## Abstract

Grade 4 adult gliomas are IDH-mutant astrocytomas and IDH-wildtype glioblastomas. They have a very high mortality rate, with survival at 5 years not exceeding 5%. We aimed to conduct a clinical imaging and morphogenetic characterization of them, as well as to identify the main negative prognostic factors that give them such aggressiveness. We conducted a ten-year retrospective study. We followed the clinical, imaging, and morphogenetic aspects of the cases. We analyzed immunohistochemical markers (IDH1, Ki-67, and nestin) and FISH tests based on the CDKN2A gene. The obtained results were analyzed using SPSS Statistics with the appropriate parameters. The clinical aspects representing negative prognostic factors were represented by patients’ comorbidities: hypertension (HR = 1.776) and diabetes mellitus/hyperglycemia (HR = 2.159). The lesions were mostly supratentorial, and the temporal lobe was the most affected. The mean volume was 88.05 cm^3^ and produced a midline shift with an average of 8.52 mm. Subtotal surgical resection was a negative prognostic factor (HR = 1.877). The proliferative index did not influence survival rate, whereas CDKN2A gene mutations were shown to have a major impact on survival. We identified the main negative prognostic factors that support the aggressiveness of grade 4 gliomas: patient comorbidities, type of surgical resection, degree of cell differentiation, and CDKN2A gene mutations.

## 1. Introduction

Formerly called glioblastomas, grade 4 gliomas of adults are represented in the latest classification of World Health Organization (WHO) by IDH-mutant grade 4 astrocytomas (the previous term used was IDH-mutant glioblastoma) and IDH-wildtype glioblastomas [1]. According to the records of the Surveillance, Epidemiology and End Results 21 program, carried out over 14 years, these entities have an incidence of 4.1 per 100,000, higher in the elderly (over 65 years) and an increased prevalence in males [2]. Their prognosis is poor, despite standard trimodal treatment (complete surgical resection and radiotherapy with concomitant and adjuvant chemotherapy) [3]. They have increased mortality, being responsible for 2.5% of cancer deaths in young adults [4]. Life expectancy at 5 years is below 5%, with an average survival rate of 14–18 months in the case of standard treatment [3].

Clinical history is usually short (3–6 months), with the symptoms being caused by direct effects on the brain parenchyma (necrosis causes motor and cognitive deficits), increasing intracranial pressure (headache), and/or tumor location (epileptic seizures) [4]. A presumptive diagnosis is performed based on the magnetic resonance imaging observation of a ring-enhanced tumor mass in T1, surrounded by hyperintensity in T2, along with an alteration of the blood–brain barrier [5]. 

Histopathological diagnosis is similar for the two entities and requires the presence of a glial proliferation associated with necrosis and/or microvascular proliferation [6]. The importance of division by the presence or absence of the IDH gene has both clinical and prognostic implications. These aspects highlight the fact that the presence of the mutation is found in younger patients and that proliferation has a less aggressive behavior [7,8]. The presence of the IDH mutation causes the hypermethylation of DNA and histone proliferation, which are some of the initial mechanisms of tumorigenesis [8]. Thus, therapeutic management is of major important, and clinical trials with chemotherapeutics and targeted vaccines on the IDH1 mutation are currently being conducted [9].

Due to the increased mortality rate of grade 4 gliomas correlated with the importance of the IDH gene, we aimed to perform a clinical imaging and morpho-genetic characterization of them, highlighting their behavior in order to identify prognostic factors for their aggression.

## 2. Materials and Methods

We conducted a retrospective study for a period of 10 years (2011–2020) of patients diagnosed with a central nervous system tumor hospitalized at the Constanta County Emergency Clinical Hospital, Dobrogea. The data were extracted from the hospital’s archives and electronic databases. The inclusion criteria were a patient age of over 18 years and a histopathological diagnosis of grade 4 glioma. The exclusion criteria consisted of recurrences. 

The clinical information and evolutionary information of the patients came from the hospitalization form. The data were evaluated by the attending physician and the neurologist. 

Imaging examinations were performed before neurosurgical intervention (computer tomography/magnetic resonance imaging) considering localization, size and volume, peritumoral edema, and midline shift. The type of resection, total or subtotal, was evaluated by tomography (CT) and/or magnetic resonance imaging (MRI) performed postoperatively. The examinations were performed at the hospital and were evaluated by the radiologist and the attending physician.

Sampled tissues were macroscopically described and prepared according to international protocols, up to the stage of microscopic slides in standard staining (hematoxylin–eosin) within the Clinical Anatomical Pathology Service of Constanta. Histopathological diagnosis was performed by two pathologists according to the latest WHO criteria (2021 edition) for the classification of tumors of the central nervous system.

The immunohistochemical examinations were performed at the Center for Research and Development of the Morphological and Genetic Studies of Malignant Pathology (CEDMOG). The evaluation was conducted by two different pathologists. Formalin-fixed and paraffin-embedded samples were sectioned at 4 µm and prepared according to the working protocol provided by the manufacturer, Master Diagnostica (Sevilla, ES).

Immunohistochemical tests used the markers IDH1 R132H (H09, ready-to-use, HIER-DAB method), Ki-67 (SP6, ready-to-use, HIER-DAB method), and nestin (10C2, ready-to-use, HIER-DAB method). The counter-staining was performed with hematoxylin–eosin. Cytoplasmic markers (IDH1 R132H and nestin) were examined throughout the section to assess the type of reactivity (positive or negative) and the intensity of the reaction (strong, moderate or weak). For the nuclear marker (Ki-67), the reference index was calculated as the percentage of positive nuclei after counting at least 1000 nuclei on 10 HPF.

CDKN2A gene alterations were performed using fluorescent in situ hybridization (FISH) at CEDMOG. The sections were made from the same formalin-fixed and paraffin-embedded samples sectioned at 3 µm. The tissue slides followed successive pre-treatment, denaturation, hybridization and post-hybridization steps according to the protocol developed by the manufacturer. The cytogenetic evaluation used ZytoLight SPEC CDKN2A/CEN 9 Dual Color Probe probes (Bremerhaven, Germany). Fluorescent signals of the preparations were calculated in 100 tumor nuclei using a Zeiss Axio Imager 2 fluorescence microscope (Zeiss Gmbh, Germany). In cells without abnormalities, two green (CDKN2A gene region) and two orange (CEN 9 probe) signals were observed. In the cells with deletion, fewer orange signals were observed, and more orange signals were observed in the case of amplification.

Statistical data analysis was performed in SPSS Statistics Version 26 (IBM Corporation, NY, USA). Indicators of central tendency and variability were used. An analysis of univariate data was performed via the chi-squared test and Fisher’s exact test for categorical data and the Mann–Whitney U test and Kruskal–Wallis H test for continuous variables. To establish the association of data, we used the Pearson correlation coefficient. Survival estimates were performed until 1 July 2022, and they were calculated using the Kaplan–Meier method. The survival differences between groups were analyzed by applying the log-rank test. Hazard ratios (HRs) were appreciated by using Cox regression analysis. Results were considered statistically significant at a *p*-value of <0.05.

All patients signed the informed consent at the time of hospitalization, and ethics approval was obtained from the local ethics commission (Ethics Commission of the Constanta County Emergency Hospital).

## 3. Results

### 3.1. Clinical Characterization

We identified 85 cases of grade 4 gliomas, 54.12% with a diagnosis of the IDH-mutant astrocytoma and 45.88% with a diagnosis of the IDH-wildtype glioblastoma. 

More than half of the cases were found in males (54.12%). The average age at the time of diagnosis was 59.05 years (20–82), with most cases being in the sixth decade of life (32.94%). In most cases, the symptoms started in the first month before hospitalization. The most common complaints were: cognitive impairment (62.35%), headache (60%) and motor deficits (60%). According to the patients’ hospitalization sheets, the most frequently reported chronic diseases were hypertension and its systemic complications (32.94%), diabetes mellitus and hyperglycemia (27.06%), and benign or malignant tumor pathologies (8.24%). Complete trimodal treatment could be performed in 75.29% of cases. The distribution of demographic aspects, symptomatology, and personal history are stratified according to diagnosis in Table 1.

**Figure 1:**
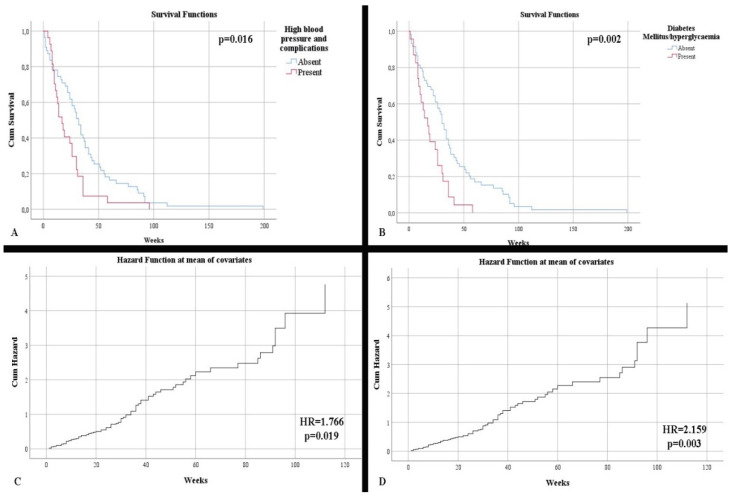
(**A**) Kaplan–Meier survival graphic that shows a lower survival rate for patients with high blood pressure. (**B**) Univariate cox regression analysis that demonstrates the risk of death in presence of high blood pressure. (**C**) Kaplan–Meier survival graphic that shows a lower survival rate for patients with diabetes or hyperglycemia. (**D**) Univariate cox regression analysis that demonstrates the risk of death in presence of diabetes mellitus or hyperglycemia.

Regarding survival, no statistically significant differences were observed concerning patient gender (*p* = 0.438). In contrast, there was an increased survival rate among patients under 50 years vs. over 50 years of age (48.5 weeks vs. 27.96 weeks, respectively) (*p* = 0.021). Neither the onset of symptoms or the symptoms influenced the survival rate. Regarding comorbidities, the absence of hypertension and its complications, as well as the absence of diabetes or hyperglycemia, were statistically correlated with higher survival rates: 38.09 weeks without hypertension and its complications vs. 22.55 weeks with hypertension and its complications; 38.20 weeks without diabetes or hyperglycemia vs. 19.56 weeks with diabetes or hyperglycemia (*p* = 0.016 and *p* = 0.002, respectively) (Figure 1A,C). Patients who completed the full treatment regimen had a superior survival rate (42.30 weeks with full completion vs. 5.91 weeks without full completion) compared with those who did not (*p* < 0.001).

Age at diagnosis was an independent risk factor for mortality (HR = 1.042, *p* = 0.001). Additionally, patients over 50 years of age were statistically shown to be in this high risk category (HR = 1.888, *p* = 0.026). The presence of hypertension and diabetes mellitus/hyperglycemia were independent risk factors for mortality (respectively, HR = 1.766, *p* = 0.019; HR = 2.159, *p* = 0.003) (Figure 1B,D).

### 3.2. Imaging Features

The main imaging technique used was CT in 92.94% of cases, with magnetic resonance imaging being performed by only 51.76% of patients. CT accuracy was 90.54%, identifying lesions with malignant features, most likely glioblastomas. Magnetic resonance imaging had a similar accuracy (90.91%).

Most gliomas were supratentorial (95.29%), with four cases located in the cerebellum. An association between symptomatology and tumor localization was noted. Supratentorial lesions were statistically significantly associated with motor deficits (*p* = 0.012), while infratentorial lesions were associated with balance and coordination disorders (*p* < 0.001). A slight predominance of lesions was observed in the left hemisphere (51.76%); however, lesions located in the right hemisphere were associated with the presence of intracranial hypertension (*p* = 0.023). The survival rate in the case of localization in the right hemisphere was higher (39.32 weeks with right hemisphere localization vs. 26.92 weeks without), though it was not a statistically significant correlation (*p* = 0.092). The temporal lobe was the site of most grade 4 gliomas (22.35%). Corroborating the data with the clinical picture, the frontally and parietally located gliomas were statistically significantly associated with the appearance of epileptic seizures and cognitive disorders (*p* < 0.001 and *p* = 0.033, respectively).

The average maximum diameter of the lesion was 51.04 mm (10–87), with approximately half of the gliomas having a diameter of over 50 mm (47.06%). The mean tumor volume was 88.05 cm^3^ (0.90–388.94). Increased tumor volume was associated with the presence of headaches and intracranial hypertension (*p* < 0.001 and *p* < 0.001, respectively). Peritumoral edema of over 5 mm was observed in 94.12% of cases. The midline shift showed an average of 8.52 mm. A higher displacement was associated with a younger age at presentation (*p* = 0.010).

**Figure 2:**
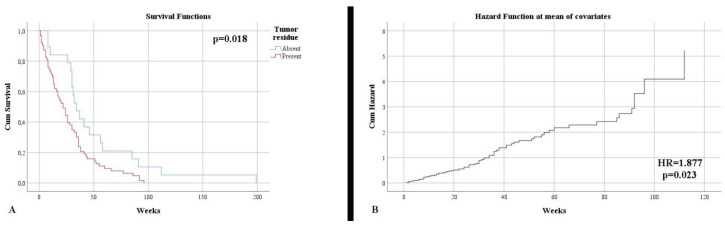
(**A**) Kaplan–Meier survival graphic that shows a lower survival rate for patients with the presence of tumor residue. (**B**) Univariate cox regression analysis that demonstrates the risk of death in presence of tumor residue.

Control imaging, performed after surgery, noted complete tumor ablation in only 23.53% of cases. This aspect was also reflected in terms of survival until the end of the study, with 96.47% of patients deceased (*p* = 0.005). The survival rate was much higher in the case of total resection (50.68 weeks) than subtotal resection (27.63 weeks) (*p* = 0.018) (Figure 2A). Additionally, the type of surgical resection was found to be an independent risk factor in terms of mortality (HR = 1.877, *p* = 0.023) (Figure 2A). The distribution of the main imaging aspects is stratified according to diagnosis in Table 2.

### 3.3. Morphogenetic Characterization

Histopathological examination supplemented with immunoreaction to IDH1 R132H showed an increased presence of IDH-mutant astrocytomas. The immunointensity of the marker was shown to be corroborated to the category of age over 50 years, as a more intense reaction was observed in people over 50 years old (*p* = 0.017).

The Ki-67 proliferation index had an average of 44.45% (4–95). A low index was associated in patients with psychiatric manifestations (*p* = 0.022), while intense proliferative activity was associated with patients with cognitive impairment (*p* < 0.001). An increased Ki-67 index was also correlated with a stronger intensity of the IDH1 immunoreaction (*p* = 0.024).

Immunoreactivity in nestin was observed in 88.24% of cases, most often being an intense reaction (58.82%). It was observed that nestin positivity was directly associated with the IDH1 immunoreaction (*p* = 0.049), and a gradually decreasing reaction was associated with a gradually increasing proliferative index (*p* = 0.001).

The FISH analysis of the CDKN2A gene showed an unaltered status in 54.12% of cases; otherwise, changes such as deletions or amplifications were found. A particular element was noted in the close association between gene amplification and the advanced age of patients (*p* = 0.001). Deletions followed by amplifications were corroborated with an increased proliferation index (*p* < 0.001). An association was also noted between the status of the gene (its mutations) and the strong immunity of nestin (*p* = 0.005). The distribution of the main morphogenetic elements is stratified according to diagnosis in Table 3.

**Figure 3:**
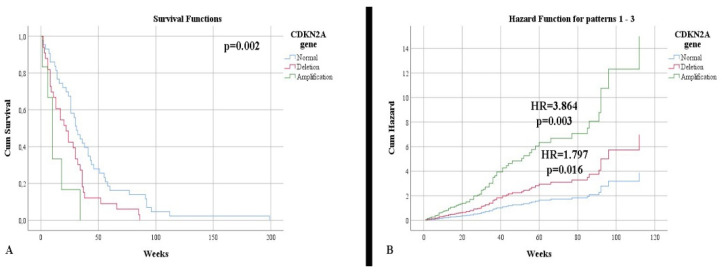
(**A**) Kaplan Meier survival graphic that shows a lower survival rate for patients with alterations of CDKN2A gene. (**B**) Univariate cox regression analysis that expresses the risk of death in presence of deletions and amplifications of CDKN2A gene.

In terms of survival, a slightly higher rate was observed in IDH-mutant astrocytomas (34.54 weeks) compared with glioblastomas (31.15 weeks), without a statistically significant correlation (*p* = 0.618). Additionally, the status of the IDH1 gene was not a risk factor for mortality (*p* = 0.625). Patients showing an immunopositivity of nestin had a lower survival rate than those without (30.86 weeks vs. 50.11 weeks, respectively), without a statistically significant correlation (*p* = 0.240). Patients with normal CDKN2A gene status had a higher life expectancy, 41.44 weeks, than patients with deletion (25.54 weeks) or amplification (13.16 weeks) (Figure 3A). After stratifying the cases according to the IDH1 gene, we noticed that the impact of altering the CDKN2A gene, associated with mortality, was higher in the case of IDH-mutant astrocytomas (*p* = 0.002). Thus, gene deletion represents an independent risk factor for mortality (HR = 1.797, *p* = 0.016), with gene amplification representing a higher mortality factor (HR = 3.864, *p* = 0.003) (Figure 3B).

## 4. Discussion

Grade 4 gliomas, IDH-mutant astrocytomas, and IDH-wildtype glioblastomas represent about half of the malignancies of the central nervous system [10]. Even though they have had a stable temporal evolution, the incidence is slightly increasing due to aging and environmental factors, whose mechanism of action is still being investigated [10,11]. This phenomenon was also identified in our study, with the age of onset being quite advanced, especially in the absence of an IDH mutation.

Clinical presentation is nonspecific and depends on the location and effects of the tumor [12]. The most common manifestations are intracranial hypertension, motor deficits, cognitive impairment, and seizures [13]. Unlike low-grade gliomas, where epilepsy may be the first symptom and is found in over 65% of cases, in the case of grade 4 gliomas, the incidence rate is low at about 25% [12,14]. This pleomorphism of the clinical picture was also noticed in our study, with no associations between symptomatology and tumor entity. The most common symptom presented by patients or identified by their relatives or the attending physician was cognitive impairment. Cognitive dysfunctions are quite common but not a concrete reason to see a doctor. Despite their increased presence, they do not influence survival and do not represent an independent factor in predicting mortality, as supported by our study (*p* = 0.407) [15]. 

In a previous study, Pierscianek D. et al. noted four independent risk factors associated with short-term survival: age, Karnofsky score, patient height and hypertension [16]. Two of these elements were also found in our study: the patient’s age (patients over the age of 50 had a lower survival rate) and the presence of high blood pressure (which is an independent predictor of mortality). The mechanism of action, especially in the elderly, is not exactly known, the action of antihypertensive drugs is speculated to occur through the production of their metabolites as neurocarcinogenetic enhancers [17].

A very important aspect noticed in both our study and a study of Montemurro N. et al. was the association of hyperglycemia with low survival, representing a risk factor for mortality. Montemurro N. et al. identified that hyperglycemia affected both overall survival and progression-free survival in 83.3% of the reviewed studies. The main mechanism of action was found to be the stimulation of the Pi3K/AKT pathway affecting the AKT/mTOR complex [18]. The Pi3K–AKT–mTOR pathway is one of the main pathways in the tumorogenesis process of both astrocytomas and glioblastomas [19].

A standard examination is conducted with magnetic resonance imaging, which can visualize weakly circumscribed intraparenchymal lesions with an infiltrative, inhomogeneous, and central hypointense character in T1 and surroundings of edema and hyperintensity in T2 [13]. In 95% of cases, localization is supratentorial (infratentorial localization is rare), as also observed in our study [4]. Most studies have shown that the preferred lobes are temporal and frontal, as in our analysis [11,20].

Unlike low-grade gliomas, where the size and volume of preoperative tumors represent negative prognostic factors, in the case of glioblastomas, this desideratum is not applicable [21,22]. This is explained by the fact that two tumors with similar dimensions can have different impacts on survival due to morphological aspects [23]. On the other hand, the analysis performed by Raj R. et al. demonstrated the opposite. Large tumors were shown to have an even better prognosis if an appropriate treatment was applied in large hospitals, such as academic centers [24]. Furthermore, the postoperative tumor volume is another prognostic factor [22,23]. In our study, the presence of tumor residue was both a negative prognostic factor (HR = 1877) and a predictor of mortality. Patients who benefited from a subtotal resection had a lower life expectancy. Thus, as in our case and the study conducted by Biju I. et al., it was noted that the total ablation of a tumor confers a longer patient survival rate [25]. In our study, a patients were provided oncological therapeutic conduct comprising radiotherapy with adjuvant and concomitant chemotherapy. Unfortunately, not everyone was able to benefit. The impact on survival was not statistically significant and was not superior to surgery (complete tumor ablation).

The latest WHO 2021 classification placed great emphasis on the status of the IDH gene. IDH1 mutations were first reported in 2008 and occur in 12% of malignant gliomas [26]. In our case, their percentage was higher, which can be explained by their progression from a lower-grade glioma [27]. In the case of the adult population, IDH mutations represent a positive prognostic factor, and they associated with a higher survival rate [26]. In a study by Brown N.F. et al., an average survival rate of 10.3 months was observed in a group of patients with IDH mutations, without the mutation being a risk factor (HR = 0.64) [28]. In our study, the survival rate was 7.94 months (34.54 weeks), and we also did not observe a risk factor for mortality (HR = 0.90). In an analysis of Liu Y.Q. et al., the average survival rate of IDH-wildtype glioblastomas, without the association of other factors, was 8.47 months, and the same rate in our study was 7.16 months (31.15 weeks) [27]. Corroborating previously presented data, a higher life expectancy was observed in the case of the presence of IDH mutation in both our study and the literature generally. 

The analysis presented in a study by Das B. et al. noted a strong association between the proliferative index and the histopathological grade of glial tumors. Additionally, some median values overlapped between consecutive grades. Thus, the utility of the Ki-67 marker is limited to differentiating a low-grade glioma from a high-grade glioma or between gliosis and a pilocytic astrocytoma [29]. In the case of grade 4 gliomas, the identification of a similar proliferation index has shown different rates of tumor progression, suggesting in the importance of morphomolecular aspects compared with proliferative ones [30]. This was also reflected in our study, in which we observed a higher mean of Ki-67 in the case of the IDH mutation, and these patients had a higher survival rate. In terms of its importance in a patient’s preoperative prognosis, Armocida D. et al. showed a directly proportional correlation between tumor size and proliferation rate [30]. We did not find the same feature (*p* = 0.520), but we observed statistically significant correlations between the proliferation rate and symptomatology (psychiatric manifestations and cognitive disorders). 

A study by Dahlort R.H. demonstrated a slight difference between the mean value of Ki-67 and the IDH mutation, without statistical significance: non-mutant grade 4 gliomas averaged 24.4% and mutant grade 4 gliomas averaged 27.5% [31]. Such a difference was also observed in our study, which is the first to identify a statistically significant correlation between the proliferative index of grade 4 gliomas, depending on the status of the IDH1 gene. At the same time, this novel finding supports the role of a positive prognostic factor in the IDH mutation, highlighted by the fact that despite an increased Ki-67 level, the survival rate was higher in this case. 

The degree of aggression quantified by Ki-67 is not relevant in the case of grade 4 gliomas, which was also supported by the reactivity to nestin. Nestin is expressed in neuroepithelial stem cells, and its activity decreases with cell maturation and differentiation [32]. It is also found in the proliferative evolution and in the metastases of other cancers such as colorectal, breast, and prostate cancers. Thus, its role in controlling proliferation and tissue renewal should be investigated in the context of the regulation of neural stem cell proliferation [32,33]. How it modulates activity is not fully understood, and so far, only the nestin–p38–EGFR pathway is known. It downregulates EGFR activity and potentiates p38, so it is associated with a more aggressive cell status because it increases its survival and replicative potential [32].

Our study demonstrated an increased proliferation rate in more differentiated cells whose nestin expression was weakly positive or negative, while undifferentiated cells (moderate or increased immunoreaction) had lower index values than precedents. This result can support the hypothesis that the activation of the nestin–p38–EGFR pathway enhances the longevity and replicative status of tumor cells. These results are also supported by the fact that the expression of nestin is correlated with the high histopathological grades of 3 or 4, suggesting tumor aggressiveness [34]. However, nestin’s role in the prognosis of patient survival is controversial. Some studies have shown that nestin is a negative prognostic factor in terms of survival, while other studies, including ours, have not identified any statistically significant correlation between its reaction and the survival of patients with grade 4 glioma (either mutant IDH or wildtype) [34,35,36,37]. 

We identified a feature, not found in the literature, that supports the aggressiveness potential of nestin immunoreactivity, corroborated with the status of the CDKN2A gene, represented by the close correlation between intensely positive reactions and the mutant status of the CDKN2A gene. The CDKN2A gene is located on chromosome 9p21.3. It has a role in the coding of two oncosuppressor proteins, p53 and pRB [38]. In grade 4 gliomas, the incidence of its mutation can be found in up to 70% of cases, and its absence leads to the activation of the gliomagenesis process by activating the Rb1 pathway [39]. The presence of deletion is more common in IDH-mutant astrocytomas (approximately 34% of cases) than in wildtype glioblastomas (approximately 28%) [39,40]. This observation was also noted in our study, suggesting the origin of IDH-mutant gliomas in low-grade precursors.

CDKN2A mutations represent a negative prognostic factor in terms of survival in both diffuse IDH-mutant and IDH-wildtype gliomas, with lower impacts on grade 2 and 3 gliomas than grade 4 gliomas [26,41,42,43]. Because of its impact on patient prognosis, we recommend the routine study of this gene so that any diffuse glial tumor IDH-mutant that possesses the deletion of the gene can be assigned as grade 4, regardless of its histopathological characteristics [43]. This recommendation is supported by the results our study, which highlights the importance of the gene and the impact of its alteration in terms of patient survival. 

The corroboration of the very low survival rate associated with CDKN2A amplifications and the association between old age and CDKN2A amplifications can be attributed to cell senescence [44]. In the case of gene deletion, the role of the p16 protein is bypassed and cells promote abnormal multiplication. In amplification, this mechanism is reversed, the cell is arrested in the cell cycle, and (along with other factors) the acquires DNA damage, which can explain the aggression we encountered in grade 4 gliomas and in malignant breast pathology [44,45].

This research was limited because we only conducted the immunohistochemical study of the IDH1 gene without analyzing IDH2 gene mutations, and we have presented no data on canonical and noncanonical alterations of the gene. It will be important to carry out studies on the influence of other aspects related to the prognosis and survival of patients (TERT-promoter mutations, EGFR amplifications, or chromosomal numerical anomalies) by comparing the two statuses of the IDH gene, given their well-known impact on wildtype IDH cases.

## 5. Conclusions

In conclusion, we succeeded in conducting a clinical imaging and morphogenetic characterization of grade 4 adult gliomas in our study, identifying the main aspects that endow them with their lethality. For their optimal management, several patient factors, the interventional technique, and the morphogenetics must be considered. The main negative risk factors that denote aggression and lead to increased mortality were identified as follows: comorbidities of patients (hypertension and diabetes mellitus or hyperglycemia), type of surgical resection, degree of cell differentiation, and alterations of the CDKN2A gene.

## Figures and Tables

**Table 1:** Stratification by the diagnosis of the demographic elements, symptomatology, and personal pathological antecedents of the patients.

Clinical Characteristics	IDH-Mutant Astrocytoma * (*n* = 46)	IDH-Wildtype Glioblastoma (*n* = 39)	*p*-Value **
Age: Average Under 50 years	57.93 (37–82) 32.61%	60.36 (20–81) 12.82%	*p* = 0.041
Gender: Male Female	56.52% 43.48%	51.28% 48.72%	*p* = 0.667
The onset of symptoms: <1 week >1 week-<1 month >1 month-<3 months	41.30% 45.65% 13.04%	38.46% 43.59% 17.95%	*p* = 0.876
Symptoms: Headache Epilepsy Motor deficits Disorders of balance and coordination Psychiatric syndromes Intracranial hypertension Cognitive impairment	60.87% 39.13% 63.04% 21.74% 19.57% 28.26% 65.22%	59.87% 30.77% 56.41% 30.77% 12.82% 46.15% 58.97%	*p* = 0.859 *p* = 0.498 *p* = 0.657 *p* = 0.457 *p* = 0.559 *p* = 0.115 *p* = 0.655
Comorbidities: High blood pressure and complications Diabetes mellitus/hyperglycemia Other tumors	26.09% 28.26% 8.70%	41.03% 25.64% 7.69%	*p* = 0.169 *p* = 0.812 *p* = 0.867
Complete treatment	69.57%	82.05	*p* = 0.215

* IDH – isocitrate dehydrogenase. ** *p*-value was assessed with chi-squared test.

**Table 2:** Stratification after diagnosis of the main aspects identified by imaging tests.

Imaging Characteristics	IDH-Mutant Astrocytoma * (*n* = 46)	IDH-Wildtype Glioblastoma (*n* = 39)	*p*-Value **
Accuracy: CT MRI	92.85% 59.47%	87.50% 92%	*p* = 0.389 *p* = 0.883
Location: Supratentorial Infratentorial	95.65% 4.35%	94.87% 5.13%	*p* = 0.627
Cerebral/cerebellar hemisphere Left Right	50% 50%	53.85% 46.15%	*p* = 0.828
Lobe Frontal Fronto-temporal Fronto-parietal Fronto-insular Parietal Parieto-temporal Parieto-occipital Temporal Temporo-occipital Temporo-insular Occipital Cerebellum	17.39% 4.35% 17.39% 2.17% 8.70% 8.70% 8.70% 23.91% 0% 2.17% 2.17% 4.35%	7.69% 0% 5.13% 7.69% 17.95% 12.82% 10.26% 20.51% 5.13% 7.69% 0% 5.13%	*p* = 0.264
Maximum diameter Average (mm) <25 mm 25–50 mm >50 mm	50.13 (10–80) 10.87% 41.30% 47.83%	52.10 (20–87) 2.56% 51.28% 46.15%	*p* = 0.704 *p* = 0.306
Volume Average (cm^3^)	85 (0.9–388.94)	91.69 (1.22–324.72)	*p* = 0.672
Resection type Total Subtotal	23.91% 76.09%	23.08% 76.92%	*p* = 0.567

* IDH – isocitrate dehydrogenase. ** *p*-value was assessed with chi-squared test, Fisher’s exact test, and Mann–Whitney U, as appropriate.

**Table 3:** Stratification after diagnosis of the main immunohistochemical and genetic aspects.

Morphogenetic Characteristics	IDH-Mutant Astrocytoma * (*n* = 46)	IDH-Wildtype Glioblastoma (*n* = 39)	*p*-Value **
Proliferative index (Ki-67) Average (%)	49.78 (15–95)	38.15 (4–90)	*p* = 0.030
Nestin: Positive Negative	82.16% 17.39%	94.87% 5.13%	*p* = 0.100
CDKN2A Normal Deletion Amplification	45.65% 47.83% 6.52%	64.10% 28.21% 7.69%	*p* = 0.173
Survival according to CDKN2A (weeks) Normal Deletion Amplification	50.74 23.96 9.67	34.08 28.73 16.67	*p* = 0.002

* IDH – isocitrate dehydrogenase. ** *p*-value was assessed with Fisher’s exact test, Mann–Whitney U, and log-rank test as appropriate.

## Data Availability

Not applicable.
